# Technik und Biomechanik der Bohr-Draht(Kirschner-Draht)-Osteosynthese bei Kindern

**DOI:** 10.1007/s00064-020-00684-6

**Published:** 2020-11-25

**Authors:** Theddy Slongo

**Affiliations:** Traumatologie des Bewegungsapparates, Kinderorthopädie, Universitätsklinik für Kinderchirurgie, Inselspital, Universitätsspital Bern, 3010 Bern, Schweiz

**Keywords:** Metaphysäre Fraktur, Epiphysäre Fraktur, Salter-Harris, Geschlossene Reposition, Offene Reposition, Metaphyseal fracture, Epiphyseal fracture, Salter-Harris, Closed reduction, Open reduction

## Abstract

**Operationsziel:**

Sichere und stabile Fixierung metaphysärer sowie epiphysärer Frakturen (Salter-Harris I–IV) mittels korrekter Bohrdraht(Kirschner[K]-Draht)-Osteosynthese, in der Folge als K‑Draht-Osteosynthese (OS) bezeichnet.

**Indikationen:**

Gemäß der AO(Arbeitsgemeinschaft für Osteosynthesefragen)-Kinderklassifikation der langen Röhrenknochen (AO Pediatric Comprehensive Classification of Long-Bone Fractures [PCCF]) alle Salter-Harris- und metaphysären Frakturen sowie Frakturen des Fuß- und Handskelettes, unabhängig von der Repositionsart, geschlossen oder offen, sofern eine Adaptationsosteosynthese eine hinreichende Stabilität zulässt. Eine K‑Draht-OS erfordert immer eine zusätzliche Fixierung/Ruhigstellung in einem Gipsverband.

**Kontraindikationen:**

Alle diaphysären Frakturen, sofern ein K‑Draht nicht im Sinne der Markraumschienung verwendet wird. Nicht korrekt reponierte respektive nicht reponierbare Frakturen.

**Operationstechnik:**

Nach geschlossener oder offener, möglichst anatomischer Reposition werden unter Durchleuchtungskontrolle 1, 2, gelegentlich 3 K-Drähte pro Fragment eingebracht. Wichtig ist dabei, dass die K‑Drähte das zu fixierende Fragment sowie das Hauptfragment (Metaphyse) optimal fassen. Es muss daher möglich sein, mit dem Durchleuchtungsgerät eine streng seitliche sowie korrekte anteroposteriore Aufnahme machen zu können. Dabei ist darauf zu achten, dass man das Gerät in die entsprechende Ebene schwenken kann. Ein Drehen der Extremität sollte auf ein Minimum beschränkt werden. Durch eine zusätzliche Manipulation zwecks Durchleuchtung könnten die zuvor optimal reponierten Fragmente erneut dislozieren. Dies wiederum kann zu einer schlechten K‑Draht-Fixierung führen. Je nach Morphologie der Fraktur, Größe der Fragmente und Lokalisation der Fraktur (Humerus, Unterarm, Femur oder Tibia, Hand oder Fuß) muss die K‑Draht-Technik angepasst werden. Diese kann sein: monolateral gekreuzt, monolateral divergierend auf- oder absteigend oder die häufigste angewendete aufsteigend gekreuzte Technik. Die K‑Drähte werden in der Regel über Hautniveau belassen und umgebogen. Somit können sie ohne erneute Narkose in der Ambulanz entfernt werden. Man muss sich bewusst sein, dass der K‑Draht weder eine Kompressions- noch eine Neutralisations-OS ist, sondern immer nur eine Adaptation. Daher braucht eine K‑Draht-OS immer eine zusätzliche Ruhigstellung mittels Gips oder konfektionierter Schiene.

**Weiterbehandlung:**

Ruhigstellung im Gipsverband für 4 bis 5 Wochen abhängig vom Alter.

**Ergebnisse:**

Bei technisch optimal durchgeführter Fixation und korrekter Indikation für eine K‑Draht-OS sowie adäquater Nachbehandlung sind die Ergebnisse sehr gut bis gut.

## Abstract (en)

**Objective:**

Safe and stable fixation of metaphyseal and epiphyseal fractures by Kirschner (K-)wire osteosynthesis. Use of various K‑wire configurations depending on the fracture morphology.

**Indications:**

In accordance with the AO Pediatric Comprehensive Classification of Long-Bone Fractures (PCCF), all Salter-Harris (SH) and metaphyseal fractures as well as fractures of the foot and hand independent of the reduction technique, closed or open, provided that adaptation osteosynthesis allows sufficient stability. Fixation/immobilization in a plaster cast is mandatory after K‑wire osteosynthesis.

**Contraindications:**

All diaphyseal fractures, if a K-wire is not used as an intramedullary nail. Fractures that can not be correctly reduced or are nonreducible fractures.

**Surgical technique:**

After closed or open, as anatomical reduction as possible, one, two, occasionally three K‑wires per fragment are inserted under fluoroscopic control. Care must be taken that the K‑wires optimally capture the fragment to be fixed as well as the main fragment (metaphysis). It must therefore be possible to make a strictly lateral and correct anteroposterior x‑ray by image intensifier. It is important that the C‑arm can be positioned at the appropriate level. Rotating the limb should be minimized, as prior to fixation the previously reduced fragments may shift again, resulting in poor K‑wire fixation. Depending on the morphology of the fracture, size of the fragments and location of the fracture (humerus, forearm, femur or tibia, hand or foot), the K‑wiring technique must be adapted, e.g., mono-laterally crossed, mono-laterally divergent, in an ascending or descending direction, or the most commonly used ascending crossed technique. In most cases, the K‑wires protrude through the sin and the exposed ends are bent. This allows removal without renewed anesthesia in the outpatient setting. K‑wire osteosynthesis is neither a compression osteosynthesis (OS) nor a neutralization OS, but is always an adaptation and fixation of the fragments. Therefore, K‑wire OS always needs additional immobilization using a plaster or prefabricated splint.

**Postoperative management:**

Immobilization in plaster cast for 4–5 weeks, depending on the age; care must be taken to avoid interference between the cast and the skin/K-wires.

**Results:**

With technically and optimally performed fixation and correct indication for K‑wire OS, as well as adequate posttreatment, very good to good results are achieved.

## Lernziele

Nach der Lektüre dieses Beitrags …

- kennen Sie die Indikationen für eine Kirschner(K)-Draht-Osteosynthese (OS),
- wissen Sie, welche Segmente des Skelettes für K‑Draht-OS geeignet sind,
- können Sie die verschiedenen K‑Draht-Konfigurationen beschreiben,
- sind Sie in der Lage, die biomechanischen Eigenschaften einer K‑Draht-OS zu erklären.

## Vorbemerkungen

Aufgrund der Morphologie des Kinderskelettes entstehen Frakturen zu einem überwiegenden Teil, bis zu 70 % [1], in den epiphysären respektive metaphysären Regionen der langen Röhrenknochen. Obwohl die Mehrzahl dieser Frakturen konservativ, also ohne interne Stabilisierung, behandelt werden kann, wird doch in gewissen Fällen eine sichere und dem kindlichen Skelett angemessene **Osteosynthese**

Osteosynthese

benötigt. Die weltweit meistverbreitete Stabilisierung epimetaphysärer Frakturen im Kindesalter erfolgt dabei mit **Bohrdrähten**

Bohrdrähten

verschiedener Dicke und mit glatter Oberfläche, sog. **Kirschner-Drähte**

Kirschner-Drähte.

Kirschner beschrieb diese OS-Technik erstmals 1909 [2, 3]. Die von ihm beschriebene Technik hat sich bis heute trotz vieler moderner Implantate und Techniken als Standard halten können.

**Figure 1:**
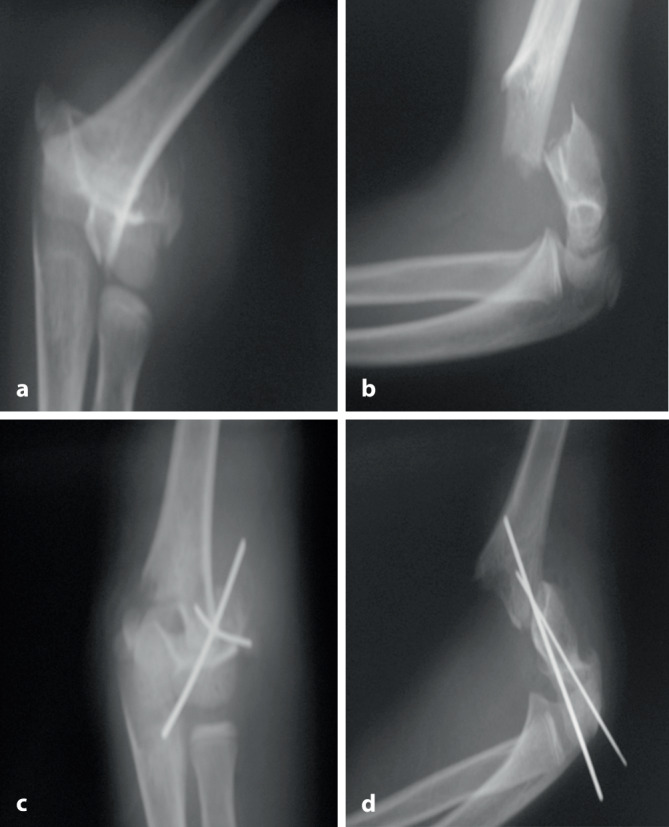

Obwohl die Kirschner-Draht-Osteosynthese prinzipiell als einfache Technik angesehen wird, sind dennoch gewisse biomechanische Gegebenheiten zu kennen und zu berücksichtigen. Zudem müssen auch die korrekten technischen Prinzipien eingehalten werden. Wie die tägliche Erfahrung jedoch zeigt, besteht hier eine nicht unwesentliche Wissens- und Handhabungslücke (Abb. 1).

Untersuchungen haben gezeigt, dass Erwachsenentraumatologen, die mehrheitlich offene Vorgehen durchführen, deutlich mehr Probleme mit dieser Fixierungstechnik haben als **Kindertraumatologen**

Kindertraumatologen,

für die diese Technik sozusagen „tägliches Brot“ ist [4]: Je öfter jemand diese Technik anwendet, desto bessere Ergebnisse werden erzielt [4, 5]. Für eine optimale Platzierung und somit auch suffiziente Stabilisierung ist jedoch primär eine perfekte, weitgehend anatomische Reposition der Fragmente essenziell. Denn es gilt der einfache Grundsatz: „Nur wenn ein K‑Draht in beiden Fragmenten fixiert ist, ist eine suffiziente Stabilität möglich.“ Somit besteht das Hauptproblem dieser Fixierungsmethode in der verbleibenden Instabilität bei **ungenügender Reposition**

ungenügender Reposition.

Dies ist bei der am häufigsten mittels K‑Draht-Osteosynthese versorgten suprakondylären Humerusfraktur exemplarisch zu beobachten. Eine ungenügende Reposition kombiniert mit daraus folgender insuffizienter Stabilisierung sind die Hauptgründe eines zunehmenden **Rotationsfehlers**

Rotationsfehlers

**Figure 2:**
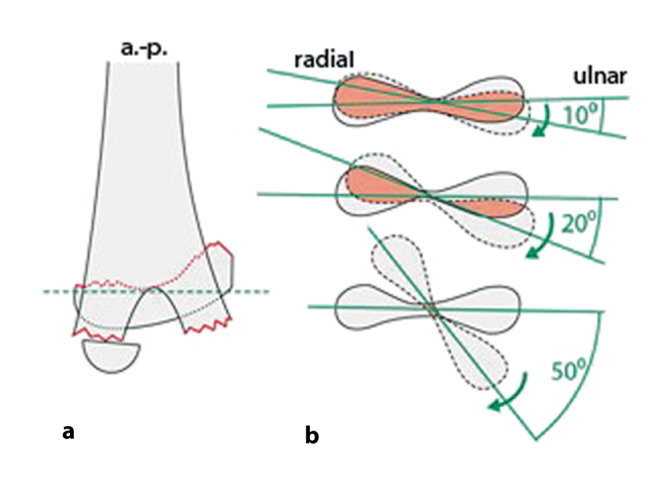

[6, 7]. Dieser Fehler „per se“ ist nicht das eigentliche Problem, sondern die damit verbundene ungenügende Auflagefläche der Fragmente, was dann zu einer Verkippung und damit verbunden zu einem unschönen „Cubitus varus“ führt (Abb. 2).

Im Weiteren ist darauf zu achten, dass aufgrund des geschlossenen Vorgehens keine iatrogenen **Nerven- und Gefäßschäden**

Nerven- und Gefäßschäden

produziert werden. Dies wiederum ist eine der häufigsten Begleitkomplikationen bei der suprakondylären Humerusfraktur.

Die in der AO(Arbeitsgemeinschaft für Osteosynthesefragen)-Kinderklassifikation beschriebene Definition der **Epimetaphyse**

**Figure 3:**
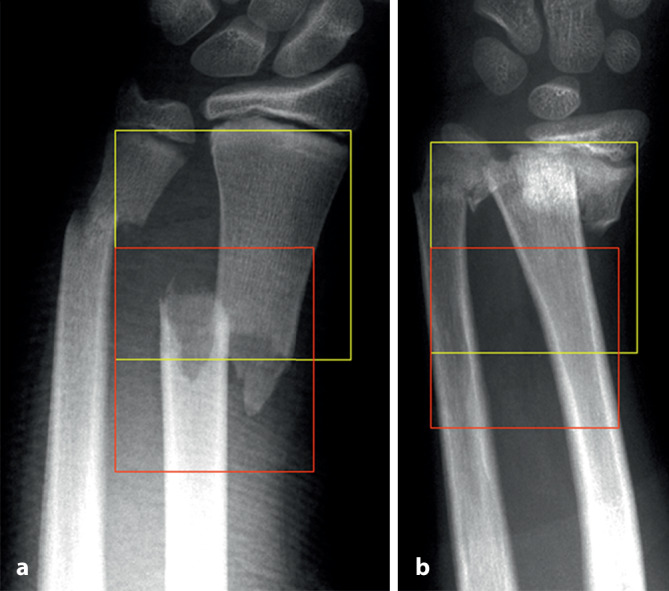

Epimetaphyse

stellt gleichzeitig das optimalste Segment für die K‑Draht-Osteosynthese dar. Es ist unbedingt darauf zu achten, dass nur Frakturen, die innerhalb des „metaphysären Quadrates“ liegenden Bereiches für diese Fixation geeignet sind. Bereits Frakturen im Bereich der Grundlinie sind mit dem Bohrdraht problematisch zu fixieren (Abb. 3; [9]).

**Figure 4:**
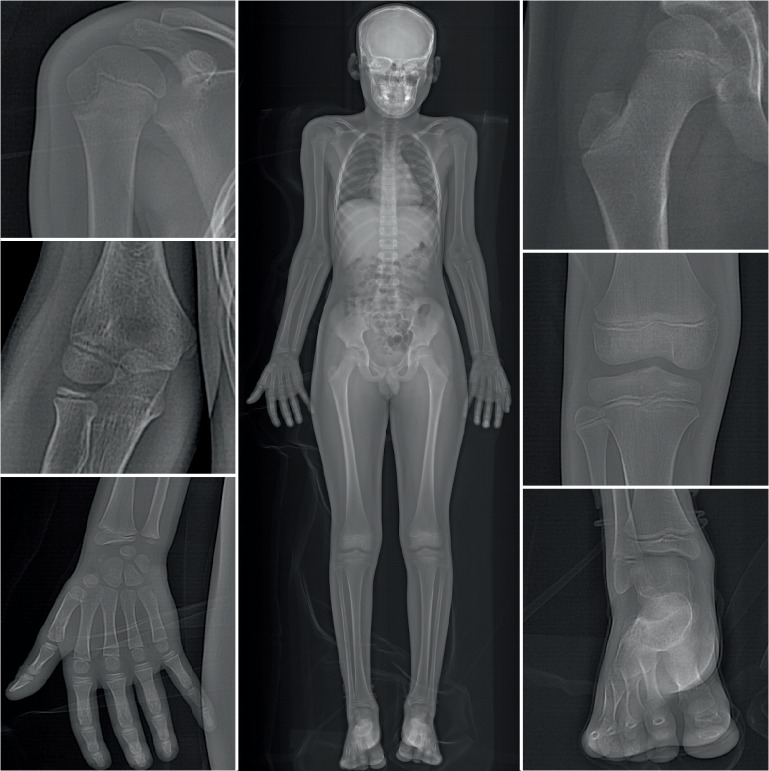

Wie in Abb. 4 dargestellt, eignen sich folgende Segmente für diese Fixationsmethode am besten:

- proximaler Humerus,
- distaler Humerus,
- distaler Radius,
- distales Femur,
- proximale Tibia,
- distale Tibia.

Ergänzend kommen Kombinationen von Fixierungen wie die **Zuggurtungsosteosynthese**

Zuggurtungsosteosynthese

der proximalen Ulna sowie Frakturen im Hand- und Fußskelett hinzu [10].

Im Folgenden möchten wir die biomechanischen Eigenschaften einer K‑Draht-OS im Generellen beschreiben sowie die unterschiedlichen Fixationstechniken, die Besonderheiten der verschiedenen oben gezeigten Segmente, vorstellen. Werden all diese erwähnten Punkte berücksichtigt, steht einer erfolgreichen K‑Draht-OS nichts mehr im Wege. Die K‑Draht-Fixation sollte in jedem Falle intraoperativ **bewegungsstabil**

bewegungsstabil

sein; sie braucht postoperativ immer eine zusätzliche Gipruhigstellung. Leider sehen wir zu oft eine ungenügende Stabilisierung, die wir gerne nur als „**betrachtungsstabil**

betrachtungsstabil

**Figure 5:**
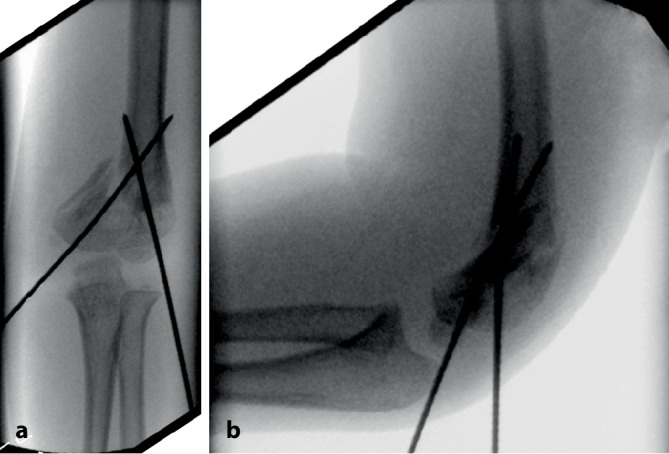

“ bezeichnen (Abb. 5). Demzufolge nennen wir solche Osteosynthesen „Frakturmanipulation mit interner Dekoration“.

## Operationsprinzip und -ziel

**Figure 6:**
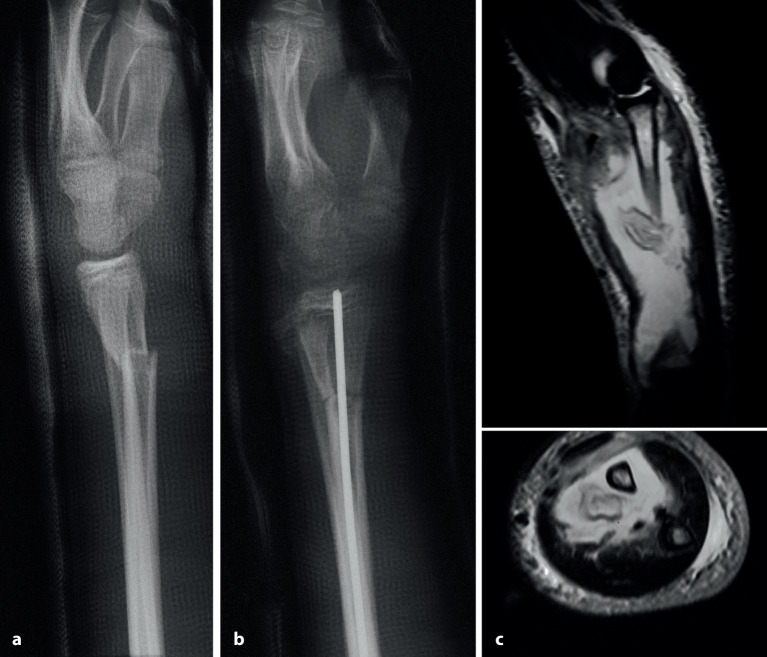

**Figure 7:**
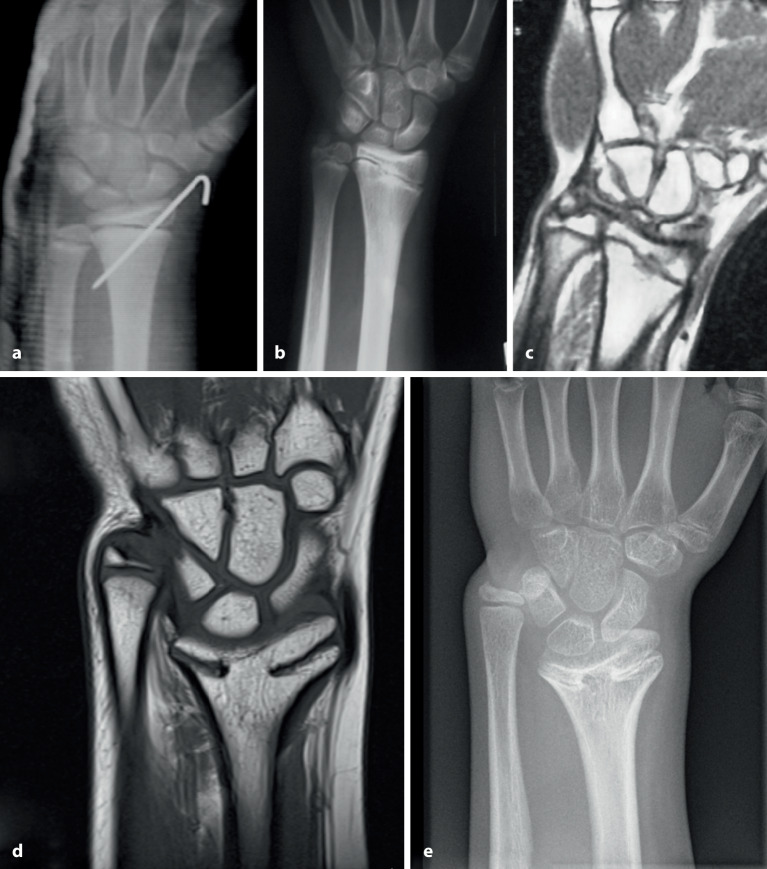

Das Operationsprinzip besteht darin, mithilfe von 2 oder maximal 3 K-Drähten, die dem Fragment und dem Alter des Kindes angepasst sein sollten, geschlossen oder offen reponierte, metaepiphysäre Frakturen zu stabilisieren [7]. Erstes und oberstes Ziel bleibt jedoch, eine solche Fraktur primär geschlossen zu reponieren und im Gipsverband ruhigzustellen. Dies setzt eine gute Handfertigkeit in der Reposition sowie dem Anlegen eines perfekten Gipsverbandes voraus. Die immer mehr vernachlässigte Schulung dieser beiden Methoden führt dazu, dass bei ungenügender Reposition schnell auf eine K‑Draht-OS gewechselt wird. Man muss sich jedoch bei jedem, auch noch so kleinen Eingriff bewusst sein, dass auch solche Eingriffe ein nicht zu vernachlässigendes Komplikationsrisiko haben, wie z. B. oberflächliche Infektionen bis hin zur Osteomyelitis (Abb. 6) oder Wachstumsstörungen bei Penetration der Fuge (Abb. 7). Daher sind die Indikationen zur Osteosynthese immer sorgfältig zu überlegen [11, 12]. Ziel einer K‑Draht-OS muss es ein, eine sichere, zumindest intraoperativ bewegungsstabile, Fixation zu erreichen.

## Vorteile

- Sicherung der Reposition
- Prävention einer sekundären Dislokation
- Dadurch anatomisch und funktionell gute Heilung

## Nachteile

- Implantatentfernung entweder in Sedation oder bei subkutan belassenen Drähten in Kurznarkose
- Pin-Track-Infektion
- Pflege der perkutan belassenen Drähte
- Gefahr der Fugenverletzung (Abb. 7)

## Indikationen

Alle Frakturen der in Abb. 4 dargestellten Skelettregionen kommen für eine K‑Draht-Fixierung infrage. Die Indikation, ob die reponierte Fraktur mittels K‑Draht stabilisiert werden muss, hängt von verschiedenen Faktoren ab:

- Alter des Kindes: Je älter das Kind, umso eher sollte eine Fraktur sicher stabilisiert werden, da das Modelling-Potenzial geringer wird,
- Größe des Fragments,
- Morphologie der Fraktur; schräg verlaufende Frakturflächen lassen sich nur schwer ohne interne Fixierung halten und gelten deshalb auch bei guter anatomischer Reposition als potenziell instabil,
- schwere Schwellungszustände, die eine alleinige externe Gipsfixierung erschweren,
- vorangegangene Repositionsversuche: Kommt es nach konservativer Behandlung zu einer sekundären Dislokation, sollte bei einer allfälligen Revision eine K‑Draht-Fixierung vorgenommen werden.

## Kontraindikationen

**Figure 8:**
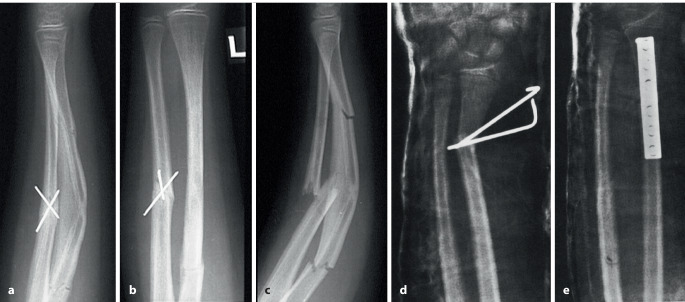

- Frakturen des metadiaphysären Übergangs (Quadrat über der Fuge der AO-Kinderklassifikation in Abb. 3)
- Diaphysäre Frakturen (sofern der K‑Draht nicht als Markraumschienung verwendet wird; Abb. 8)
- Stabile Frakturen

## Patientenaufklärung

- Offene Aufklärung der Eltern/des Kindes über alle möglichen Behandlungsverfahren inklusive Verwendung eines externen Fixateurs
- Verfahrenswechsel von geschlossener auf offene Reposition
- Allgemeine Operationsrisiken
- Postoperative Pflege der perkutanen K‑Draht-Eintrittsstellen
- Pflege des Gipsverbandes
- Mögliche residuelle Fehlstellungen oder Fehlfunktionen
- Heilungsdauer
- Metallentfernung
- Physiotherapie nur in Ausnahmefällen

## Operationsvorbereitungen

**Figure 9:**
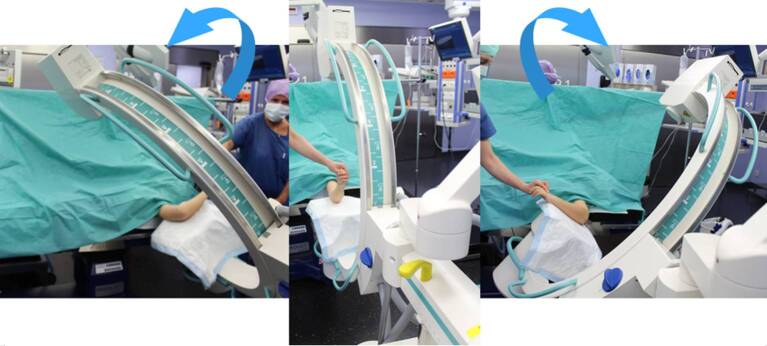

- Aktuelles Unfallröntgenbild in 2 Ebenen: Man muss sich jedoch bewusst sein, dass solche Bilder immer nur „Momentaufnahmen“ sind und dass durch jede Manipulation, besonders unter Narkose, sich die Fraktur anders darstellen kann.
- Bei unklaren Situationen bezüglich Reponierbarkeit und Stabilisierung immer in Operationsbereitschaft arbeiten; d. h. nicht nur im Gipsraum reponieren. Dadurch können kritische Situationen umgangen respektive Kompromisse vermieden werden. Damit ist gemeint, dass man schlechte Stellungen oder ungenügende Stabilität nicht akzeptieren sollte, nur weil man die Möglichkeit zur K‑Draht-Fixation nicht hat.
- Durchleuchtungsmöglichkeit (Abb. 9).
- Besprechung mit der Anästhesie bezüglich Narkoseart; Relaxation erleichtert das Reponieren.
- Genaue Analyse der Frakturmorphologie.
- In Abhängigkeit der Frakturplanung, der Bohrdrahtlage und -richtung.
- Information des Operationspersonals über die geplante Art der Behandlung.

## Instrumentarium und Implantate (Abb. 10)

- K‑Drähte 1,6 mm oder 2,0 mm für die obere Extremität; 2,5 mm oder 3,0 mm für untere Extremität. Prinzipiell muss die Bohrdrahtdicke jedoch dem Alter und der Fragmentgröße angepasst sein
- Bohrmaschine
- Gipsmaterial

**Figure 10:**
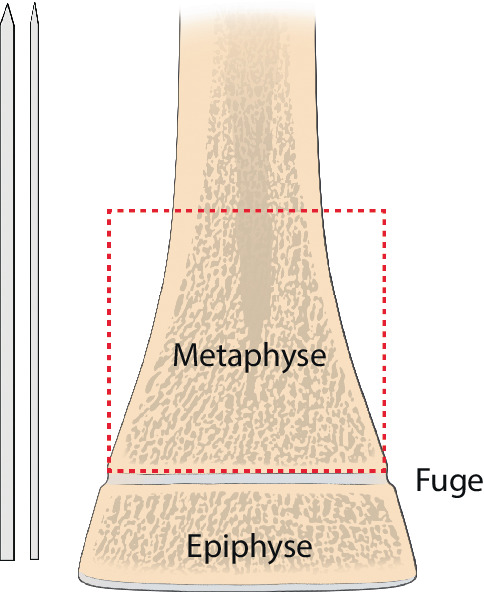

## Anästhesie und Lagerung

**Figure 11:**
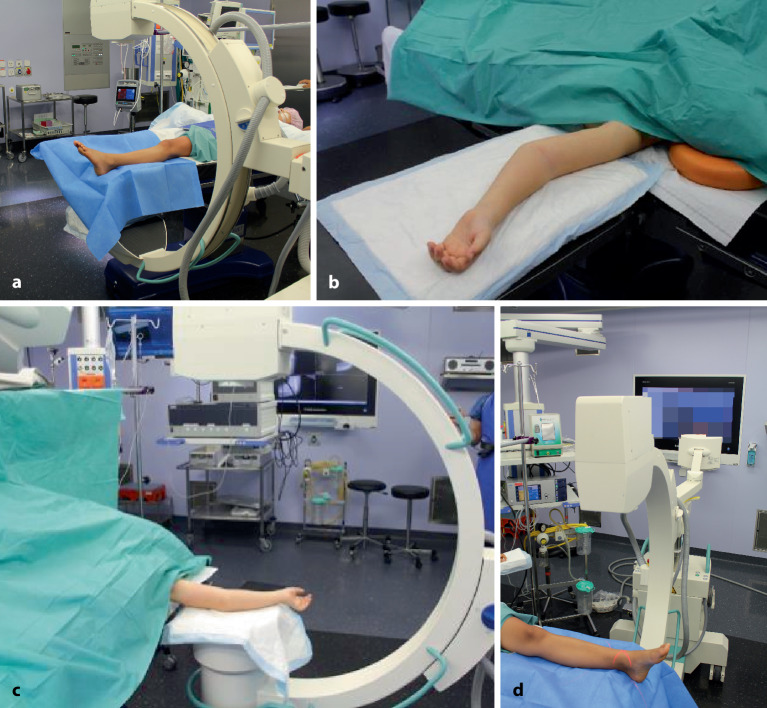

- Intubationsnarkose; Relaxation erleichtert die Reposition, besonders für die untere Extremität.
- In der Regel normale Rückenlage, dies gilt für die obere wie auch untere Extremität.
- Röntgenstrahldurchlässiger Armtisch respektive Operationstisch mit freier Durchleuchtungsmöglichkeit (Abb. 11a, b):
  - Standardmäßig wird die Extremität auf dem röntgenstrahldurchlässigen Arm‑/Operationstisch gelagert.
    Es ist wichtig, vor Beginn der Reposition zu prüfen, ob der Bildverstärker frei unter dem Hand- oder Operationstisch bewegt werden kann und damit die erforderliche Position erzielt wird.
  - Der Laser am Strahler zur strahlungsfreien Positionierung des C‑Arms, gepulstes Röntgen und die maximale Einblendung der Schlitz- bzw. Irisblende sind gefordert.
  - Wird die Extremität ausnahmsweise direkt auf den steril abgedeckten Bildwandler (Abb. 11c, d; [13]) gelagert (Berner Schule), kann der Laser nicht verwendet werden, da er abgedeckt ist. Die Zentrierung auf die Fraktur erfolgt unter Röntgenstrahlung. Dabei muss jedoch eine höhere Strahlenbestrahlung in Kauf genommen werden, die jedoch durch die kürzere Durchleuchtungszeit beim erfahrenen Operateur und bei höherer Bildqualität deutlich kompensiert wird. Über längere Röntgenzeit beim weniger Erfahrenen wird in der Literatur berichtet.

## Biomechanik von Kirschner-Drähten

Bei der Verwendung von Kirschner-Drähten muss man sich immer bewusst sein, dass es sich dabei um eine **Adaptationsosteosynthese**

Adaptationsosteosynthese

handelt und niemals um eine Kompressions- oder Neutralisationsosteosynthese, wie sie Schrauben oder Platten sind. Es geht darum, die Fragmente in einer möglichst optimalen Weise stabil zueinander zu halten. Deshalb ist praktisch immer additiv eine zusätzliche **Gipsruhigstellung**

Gipsruhigstellung

vorzunehmen.

**Figure 12:**
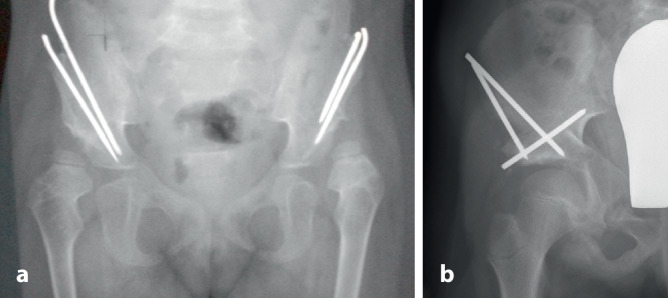

Selbst im Gipsverband können sich K‑Drähte durch die Mikrobewegungen und größeren Bewegungen im Gips auslockern. Deshalb ist der Anordnung der K‑Drähte besondere Aufmerksamkeit zu schenken. Leider wird diesem Aspekt zu oft nicht Rechnung getragen; davon zeugen die doch recht häufigen sekundären Fehlstellungen trotz K‑Draht-Fixierung. Dabei sind die beiden häufigsten beobachteten Fehler:

- parallel eingebrachte Drähte,
- zu nahe beieinanderliegende Drähte, die eigentlich nur die Wirkung eines einzelnen Drahtes haben (Abb. 12a, b).

Die optimale **Anordnung von K‑Drähten**

Anordnung von K‑Drähten

ist deshalb, je nachdem ob diese nur von einer Seite oder beidseitig eingebracht werden,

- auf- oder absteigend gekreuzt,
- monolateral gekreuzt,
- monolateral divergierend,

wobei immer darauf geachtet werden muss, dass die jeweiligen Kreuzungsstellen nicht auf Frakturhöhe liegen respektive bei divergierender Technik außerhalb des Knochens (Abb. 17 und 28).

### Einfluss der Kirschner-Draht-Stärke

Der **Durchmesser**

**Figure 13:**
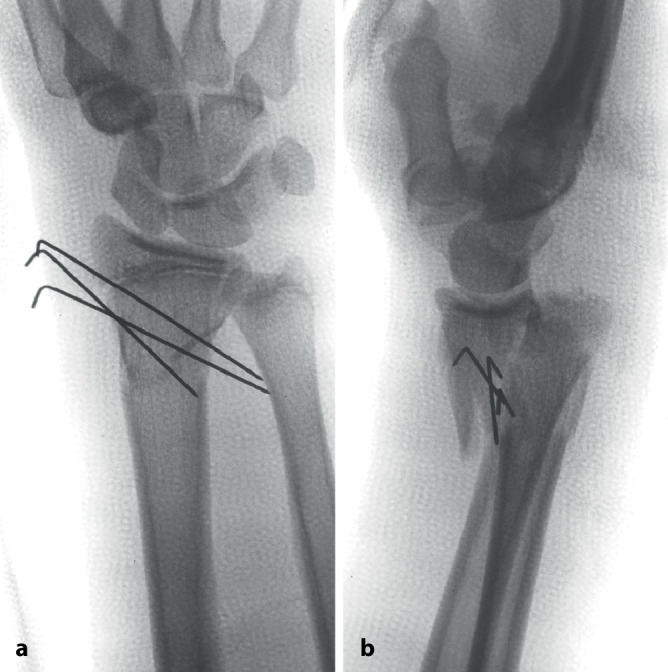

Durchmesser

des K‑Drahtes hat einen hohen Einfluss auf die Stabilität. Dabei steht man jedoch im Konflikt mit möglicher Schädigung der Wachstumsfuge, sofern diese gekreuzt werden muss. In diesem Fall ist ein mehrmaliges Bohren unbedingt zu vermeiden. Es ist somit ratsamer, einen etwas dickeren Bohrdraht zu nehmen, der sich präzise zielen und einbringen lässt, als zu feine Bohrdrähte, die dann ungünstig liegen und wiederholt eingebohrt werden müssen (Abb. 13).

Die Tab. 1 zeigt die Relation der am häufigsten gebrauchten K‑Draht-Durchmesser zu dessen Fläche: Als Wert 100 % haben wir den Ø 1,6 mm genommen.

**Table 1:** 

Drahtdurchmesser	Fläche	Verhältnis zu 2,0 mm Draht	Verhältnis zu 1,6 mm Draht
[mm]	[mm^2^]	[–]	[–]
1	0,785	0,250	0,391
1,2	1,131	0,360	0,563
1,6	2,011	0,640	1,000
2	3,142	1,000	1,563
2,5	4,909	1,563	2,441
3	7,069	2,250	3,516

Somit hat ein 2,0-mm-K-Draht eine 25 % höhere Zugfestigkeit als einer von 1,6 mm.

Für die **Beugefestigkeit**

Beugefestigkeit

hingegen, die sich aus der 3. Potenz des Radius berechnen lässt, ist der Einfluss des Durchmessers von noch größerer Bedeutung. Wie in Tab. 2 zu sehen ist, ist der Unterschied in der Festigkeit zwischen 1,6 und 2,0 mm praktisch 100 %, zwischen 1,6 und 3,0 mm mehr als 600 %.

**Table 2:** 

Drahtdurchmesser	Widerstandsmoment gegen Biegung	Verhältnis zu 2,0 mm Draht	Verhältnis zu 1,6 mm Draht
[mm]	[mm^3^]	[–]	[–]
1	0,098	0,125	0,244
1,2	0,170	0,216	0,422
1,6	0,402	0,512	1,000
2	0,785	1,000	1,953
2,5	1,534	1,953	3,815
3	2,651	3,375	6,592

### Einfluss der Kirschner-Draht-Spitze

Die **Geometrie**

Geometrie

der K‑Draht-Spitze hat einen wesentlichen Einfluss auf das Einbringen des Drahtes. Die meistverbreitete Spitzenart ist die **Trokarspitze**

Trokarspitze.

**Figure 14:**
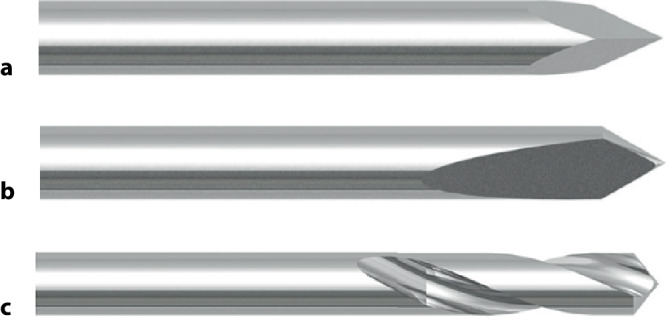

Diese hat eine 3‑eckige (oder gelegentlich 4‑eckige) Form, die nur knapp geschliffen und nicht anderweitig speziell geformt ist. Sie gleicht somit praktisch einer normalen Nagelspitze mit 3 Flächen (Abb. 14a). Diese Spitzenform erschwert das sehr tangentiale Einbohren des Drahtes. Deshalb sollte man immer zuerst weitgehend rechtwinklig zur Knochenoberfläche mit dem Bohren beginnen und erst, wenn eine genügende Vertiefung respektive ein Loch entstanden ist, den Draht tangential absenken. Da diese Spitze auch nicht sehr scharf ist, erzeugt sie auch sehr viel Hitze. Deshalb sollte man während des Einbohrens mit Wasser kühlen und/oder oszillierend bohren.

Daneben finden wir auch verbreitet die sog. **Bajonettspitze**

Bajonettspitze;

diese zeichnet sich durch einen einseitigen, flächigen Schliff aus und ist bedeutend schärfer als die Trokarspitze; sie ist auch als „Ilizarov-Bohrdraht-Spitze“ bekannt (Abb. 14b).

Zu diesen Spitzenformen bieten einige Hersteller auch Bohrdrähte mit einer 2‑spiraligen, sehr kurzen **bohrerähnlichen Spitze**

bohrerähnlichen Spitze

an (Abb. 14c).

### Einfluss der Kirschner-Draht-Ausrichtung auf die Stabilität

**Figure 15:**
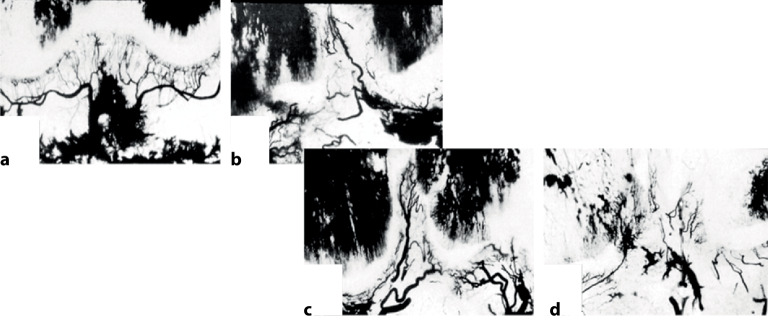

Da, wie bereits erwähnt, mit den K‑Drähten keine Kompression erzeugt werden kann, ist die Anordnung respektive die Ausrichtung der K‑Drähte von entscheidender Bedeutung. Werden die K‑Drähte korrekt gesetzt, kann eine relative Stabilität erreicht werden, die das Verschieben der Fragmente verhindert. In den nachfolgenden Grafiken soll dies bildlich veranschaulicht werden (Abb. 15 und 16**;** [14]).

**Figure 16:**
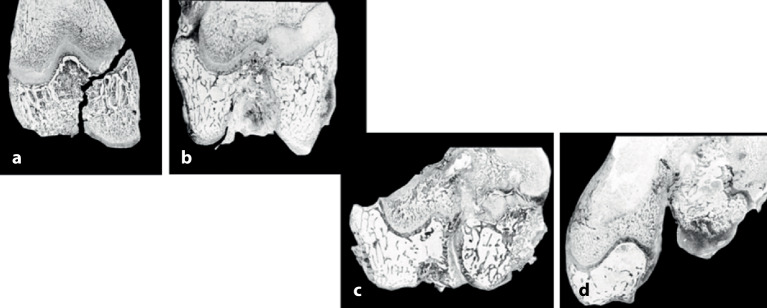

## Operationstechnik

Abb. 17, 18, 19, 20, 21, 22, 23, 24, 25, 26, 27, 28, 29

**Figure 17:**
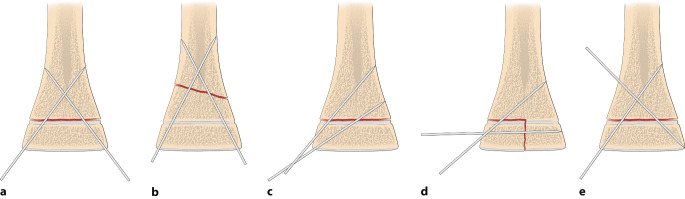

**Figure 18:**
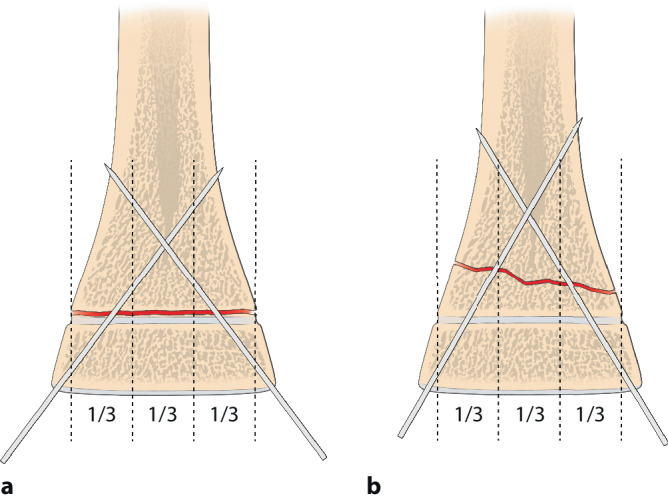

**Figure 19:**
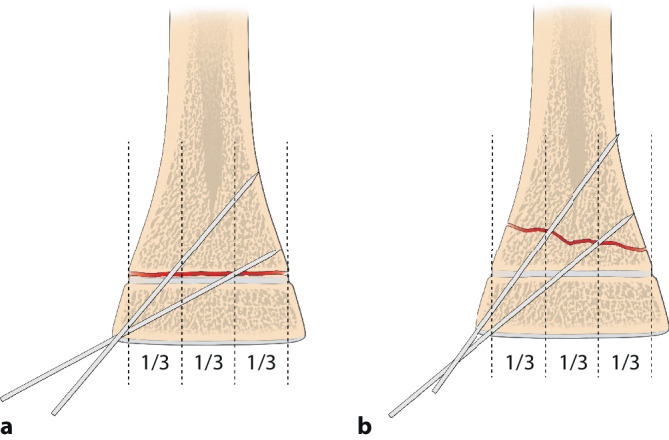

**Figure 20:**
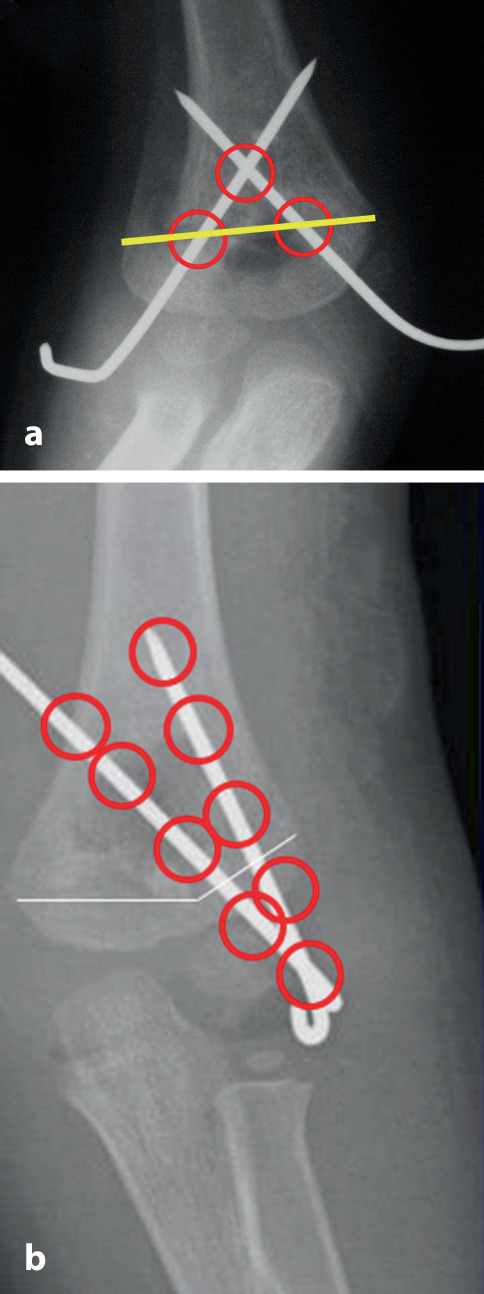

**Figure 21:**
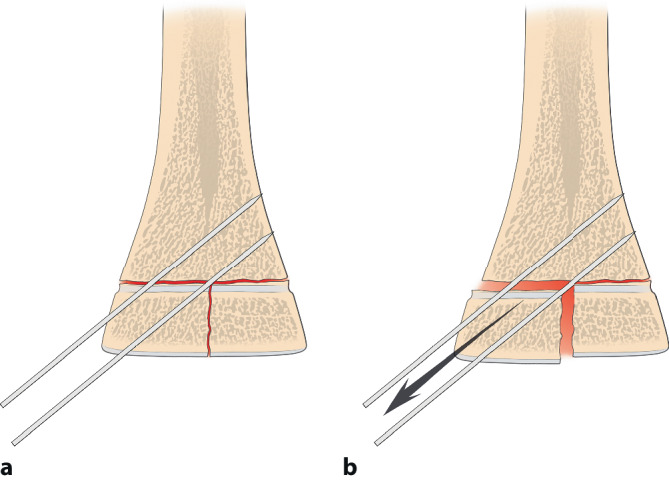

**Figure 22:**
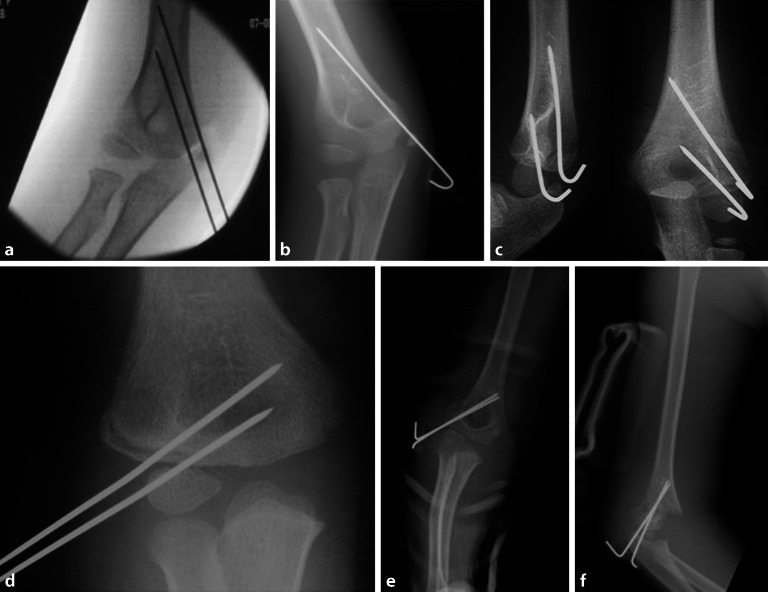

**Figure 23:**
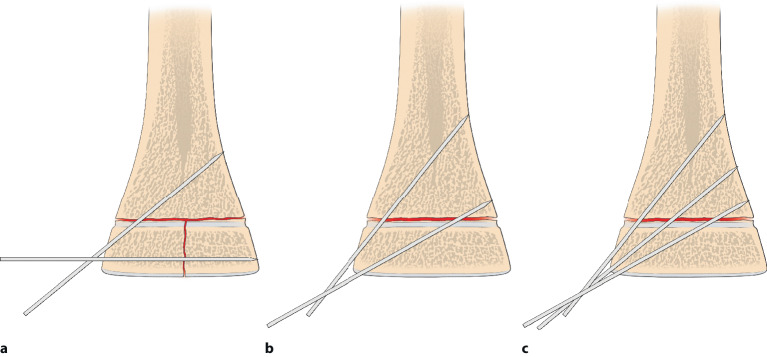

**Figure 24:**
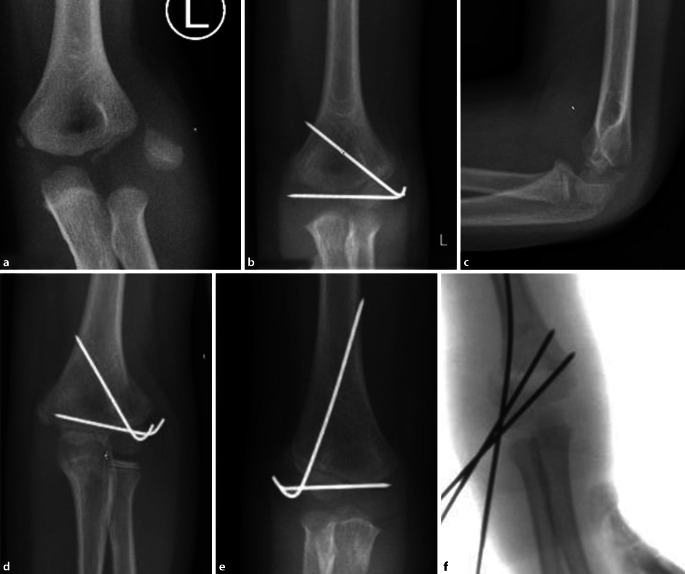

**Figure 25:**
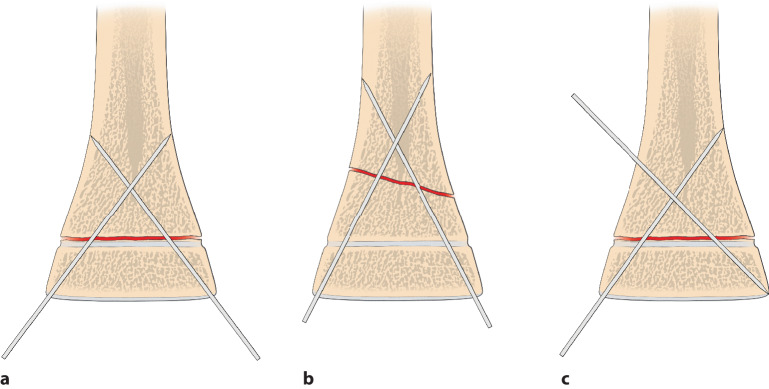

**Figure 26:**
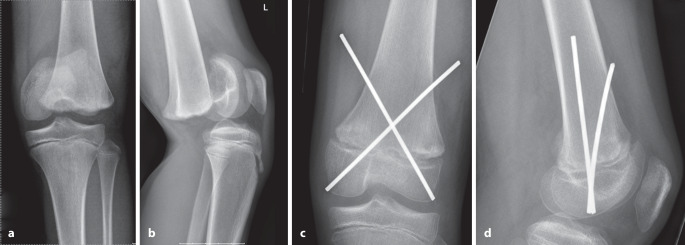

**Figure 27:**
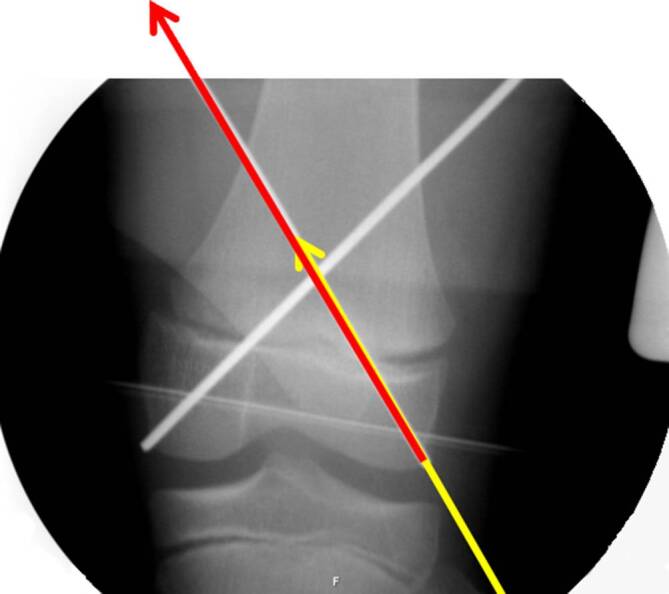

**Figure 28:**
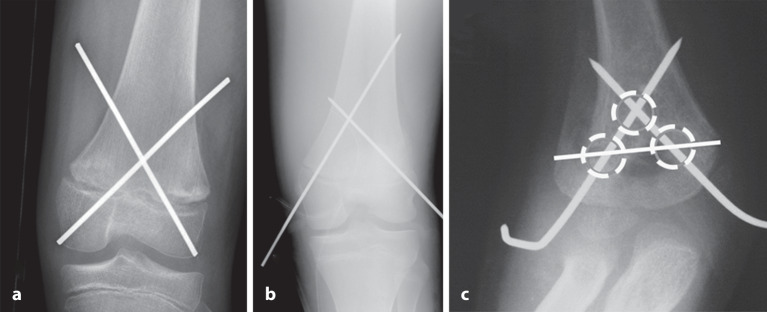

**Figure 29:**
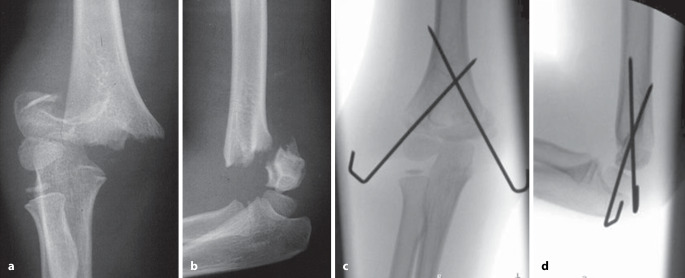

## Postoperative Behandlung

Wie bereits anfangs erwähnt, ist die Kirschner-Draht-Osteosynthese nicht belastungsstabil, sollte jedoch in jedem Falle lagerungs- bis bewegungsstabil sein je nach Lokalisation der Fraktur, Größe der fixierten Fragmente sowie Alter des Kindes. Deshalb sind postoperativ folgende Punkte zu beachten:

- gute, wenn möglich noch intraoperative, gipsfreie Röntgendokumentation,
- zusätzliche Ruhigstellung in Gipsschiene (einfach oder doppelseitig) oder in zirkulärem Gipsverband. Gelegentlich kommen auch kommerziell erhältliche Produkte zur Anwendung,
- an der unteren Extremität vorzugsweise zirkuläre Verbände, gespalten,
- über die Haut hinausragende (perkutan eingebrachte) K‑Drähte sollten den Gips nicht berühren, deshalb
- immer Gipsfenster um den K‑Draht herum,
- Anlernen der Eltern für die Gips- allfällig K-Draht-Pflege,
- Hospitalisation je nach Schwere des Traumas oder gemäß klinikinternen Vorgaben, meist 1 bis 3 Tage,
- erste klinische und radiologische Kontrolle bei Kindern bis 4/5 Jahren nach 4 Wochen, bei älteren Kindern nach 5 Wochen,
- unter leichter Sedation oder mit Schmerzmitteln ambulante Entfernung der Kirschner-Drähte, sofern perkutan eingebracht, ansonsten Planung für Entfernung in Kurznarkose,
- weitere Nachkontrolle 3 Monate postoperativ zwecks funktioneller Prüfung der Beweglichkeit, da es sich immer um gelenknahe Verletzungen handelt.

## Fehler, Gefahren, Komplikationen

Auf die Fehler einer K‑Draht-Osteosynthese wurde anhand der Abbildungen und Abbildungstexte schon mehrmals hingewiesen. Zusammenfassend sollen nochmals hervorgehoben werden:

- falsche Indikation (Fraktur außerhalb des metaphysären Quadrates, Abb. 3),
- keine optimale respektive korrekte Reposition der Fragmente vor der OS,
- Nicht-Fassen der Fragmente,
- falsches oder suboptimales Einbohren der K‑Drähte,
- suboptimale K‑Draht-Dicke,
- biomechanisch nicht korrekte Anordnung der K‑Drähte (gekreuzt oder monolateral divergierend),
- Nicht-Beachtung der Drittel- respektive Viertel-Regel,
- die Gefahren der K‑Draht-OS sind v. a. der Repositionsverlust mit allfälligem anatomisch wie funktionell schlechtem Ergebnis,
- Gefahr einer Nerven- oder Gefäßschädigung, besonders am Ellbogen zu beachten,
- die Kombination Gipsverband und perkutan herausragende K‑Drähte bringt immer die Gefahr einer oberflächlichen, im schlimmsten Fall tiefen Infektion mit sich,
- unabhängig vom fixierten Fragment respektive von der fixierten Fraktur ist ein Repositionsverlust mit entsprechender anatomischer Fehlstellung respektive Funktionseinbuße immer als schwerwiegende Komplikation anzusehen,
- Drahtbruch,
- Auswandern des Drahtes.

## Ergebnisse

Unter Berücksichtigung der Anwendungshäufigkeit der K‑Draht-Osteosynthese wie vorgängig beschrieben, nicht grob fahrlässige Fehler begangen werden, sind die Resultate dieser Methode als sehr gut anzusehen. Das größte Problem besteht darin, dass man diese OS als zu einfach ansieht und sich die wichtigsten Punkte einer optimalen Fixation nicht immer wieder vor Augen führt. Im Weiteren werden zu gravierende, meist auch radiologisch dokumentierte Fehlstellungen akzeptiert unter der Annahme, dass das kindliche Skelett dies schon „Ausbügeln“ wird [11, 15, 16, 17].

## CME-Fragebogen

In welchem Segment sind Frakturen des kindlichen Skeletts überwiegend lokalisiert?

Schaft langer Röhrenknochen

Epi-/metaphysäre Region langer Röhrenknochen

Schaft kurzer Röhrenknochen

Epi-/metaphysäre Region kurzer Röhrenknochen

Kurze Knochen (Ossa brevia)

Was ist das wesentliche, primäre Problem einer *un*genügenden Frakturreposition einer suprakondylären Humerusfraktur mit verbleibender Instabilität?

Rotationsfehler

Ungenügende Auflagefläche der Fragmente

Fragmentverkippung

Cubitus carus

Fehlende Bewegungstabilität

In welchem Bereich sollte eine Fraktur lokalisiert sein, um die Indikation zu einer K‑Draht Osteosynthese zu stellen?

im metaphysären Quadrat

im epiphysären Rechteck

im diaphysären Viereck

mind. 2 Querfinger proximal der Epiphysenfuge

in der Epiphyse

Was soll mit einer K‑Draht Fixation erreicht werden?

Absolute Stabilität

Übungsstabilität

Bewegungsstabilität

Adaptation der Fragmente und sichere Bewegungsstabilität

Kompression

Welches der genannten postoperativen Risiken ist bei der geschlossenen Versorgung einer suprakondylären Humerusfraktur eine häufige Begleitkomplikation?

Nachblutung mit ausgeprägter Hämatombildung

bleibende Bewegungseinschränkung

Rotationsfehler

iatrogener Nervenschaden

Gestörter Längenwachstum

Welche der genannten Regionen ist *am wenigsten* für eine K‑Draht-Osteosynthese geeignet?

proximaler Humerus

distaler Humerus

proximaler Radius

distaler Radius

distales Femur

Was ist der *Nachteil* der Lagerung einer Extremität direkt auf der Bildwandlerkamera?

Die Bildqualität ist schlechter

Die Durchleuchtungszeit ist länger

Die Lagerung ist weniger stabil

Das BV Gerät kann beschädigt werden

Die Streustrahlung ist etwas grösser, diese wird jedoch durch die geringere Durchleuchtungszeit kompensiert.

Was wird durch den zunehmenden K‑Draht Durchmesser am stärksten beeinflusst?

Das präzisere Platzieren

Die Zugfestigkeit

Die Beugefestigkeit

Die Rotationsstabilität

Die Drahtausrichtung

Wie sollten K‑Drähte eingebracht werden, damit sie das Fragment bestmöglich fixieren?

Möglichst parallel

Drei bis vier K‑Drähte sind immer besser, unabhängig wie sie eingebracht wurden

In der Regel genügen zwei, sofern sie optimal gekreuzt oder genügend divergierend platziert werden, die Kreuzungsstelle sollte nie auf Frakturhöhe sein

Sie sollten sich auf der Gegenkortikalis treffen

Es sollen möglichst dünne K‑Drähte verwendet werden, um die Fugen nicht zu verletzen

Was besagt resp. beschreibt die „Drittel-Regel“?

Es sollten, wenn möglich, immer 3 K-Drähte eingebohrt werden

Knochendurchmesser zu Fragmentgrösse sollte nie kleiner als 1 zu 3 sein

Die Überkreuzung der K‑Drähte auf Höhe der Fraktur sollte, wenn möglich beidseitig, am Übergang vom äusseren zum mittleren Drittel der Frakturlinie sein.

Ich kenne diese Regel nicht, noch nie gelesen

Diese Regel besagt, dass ein Drittel aller mit K‑Draht fixierten Frakturen potenziell einen Repositionsverlust erleiden.
